# AadA36, a novel chromosomal aminoglycoside nucleotidyltransferase from a clinical isolate of *Providencia stuartii*

**DOI:** 10.3389/fmicb.2022.1035651

**Published:** 2022-11-01

**Authors:** Mengdi Gao, Chunlin Feng, Yongan Ji, Yaokai Shi, Weina Shi, Lei Zhang, Shuang Liu, Anqi Li, Xueya Zhang, Qiaoling Li, Junwan Lu, Qiyu Bao, Hailin Zhang

**Affiliations:** ^1^Department of Children’s Respiration Disease, The Second Affiliated Hospital and Yuying Children’s Hospital, Wenzhou Medical University, Wenzhou, China; ^2^Key Laboratory of Medical Genetics of Zhejiang Province, Key Laboratory of Laboratory Medicine, Ministry of Education, China, School of Laboratory Medicine and Life Sciences, Wenzhou Medical University, Wenzhou, China; ^3^Medical Molecular Biology Laboratory, School of Medicine, Jinhua Polytechnic, Jinhua, China

**Keywords:** AadA36, *Providencia stuartii*, aminoglycoside nucleotidyltransferase, novel aminoglycoside resistance gene, kinetic analysis

## Abstract

In this study, we characterized a novel chromosome-encoded aminoglycoside nucleotidyltransferase (ANT), AadA36, from the *Providencia stuartii* strain P14 isolated from the sputum specimen of a burn patient at a hospital in Wenzhou, China. Among the functionally characterized ANTs, AadA36 shared the highest amino acid sequence identity of 51.91% with AadA14. The whole genome of *P. stuartii* P14 consisted of one chromosome and two plasmids (designated pP14-166 and pP14-114). A total of 19 genes with ≥80% similarity with functionally characterized antimicrobial resistance genes (ARGs) were identified in the whole genome, including aminoglycosides [*aac(2′)-Ia*, *aph(6)-Id*, *aph(3″)-Ib*, *aac(6′)-Ib*, *ant(3″)-IIa*, *aph(3′)-Ia*], β-lactams (*bla*_CMY-2_ and *bla*_OXA-10_) and so on. Antimicrobial susceptibility testing showed that the *aadA36* gene conferred specific resistance to spectinomycin and streptomycin, and the minimum inhibitory concentration (MIC) of these antimicrobials increased 128- and 64-fold compared with the control strain. The kinetic parameters of AadA36 were consistent with the MIC data of spectinomycin and streptomycin, with *k_cat_*/*K_m_* ratios of (1.07 ± 2.23) × 10^4^ M^−1^ s^−1^ and (8.96 ± 1.01) × 10^3^ M^−1^ s^−1^, respectively. The identification of a novel aminoglycoside resistance gene will help us further understand the complexity of the resistance mechanisms and provide deep insights into the dissemination of resistance genes in the microbial population.

## Introduction

Aminoglycoside antimicrobials are broad-spectrum agents with strong antibacterial effects that are used for the treatment of bacterial infections, especially those caused by Gram-negative bacilli, including *Escherichia coli*, *Klebsiella pneumoniae*, *Klebsiella oxytoca*, *Enterobacter cloacae*, *Enterobacter aerogenes*, *Providencia* spp., *Proteus* spp., *Morganella* spp., and *Serratia* spp. (Krause et al., 2016). This class of antimicrobials exerts bactericidal effects mainly by interfering with bacterial protein synthesis. Moreover, these antimicrobials can also be combined with other classes of antimicrobials to treat severe infections. In recent years, the resistance of Gram-negative bacilli to aminoglycosides has become increasingly severe. In the clinical setting, the most common mechanism of resistance to the aminoglycoside (AG) antimicrobials is the enzymatic modification (Wright, 1999; Ramirez and Tolmasky, 2010; Labby and Garneau-Tsodikova, 2013). According to their modification ability, the aminoglycoside-modifying enzymes (AMEs) are divided into three main groups: aminoglycoside acetyltransferases (AACs), aminoglycoside phosphotransferases (APHs), and aminoglycoside nucleotidyltransferases or adenyltransferases (ANTs or AADs; Ramirez and Tolmasky, 2010).

The ANT group can be further divided into five subtypes based on the specific position adenylated by the enzymes on aminoglycosides, including ANT(6), ANT(9), ANT(4′), ANT(3″), and ANT(2″). To date, more than 30 types of ANT(3″) enzymes have been described in the Comprehensive Antibiotic Resistance Database (CARD),^1^ designated AadA1 to AadA31 with some numbers missing (Ramirez and Tolmasky, 2010). The ANT (3″) enzymes are the most common ANTs and include two subclasses [ANT (3″)-I, ANT (3″)-II] that confer specific resistance to streptomycin and spectinomycin based on the mechanism of adenylation on the 3′′- and 9-hydroxyl groups of streptomycin and spectinomycin, respectively (Shaw et al., 1993). Moreover, the genes encoding ANT (3″)-Ia proteins are most commonly named *aadA* (Hollingshead and Vapnek, 1985). Correspondingly, the proteins encoded by *aadA* genes are designated AadA.

Bacteria of the genus *Providencia* in the family *Morganellaceae* are Gram-negative opportunistic pathogens. The *Providencia* group originated from the paracolon bacterial strain 29911, which was discovered in 1943 (Ewing et al., 1954; Hawkey, 1984). This genus experienced considerable taxonomic instability from 1952 to 1962 because the *Providencia* strains appeared to be an intermediate group between *Proteus morganii* and *Proteus rettgeri*. *Providencia stuartii* was named in 1962 and was further verified based on DNA–DNA hybridization in 1978 (O'Hara et al., 2000). *P. stuartii* is one of the most common pathogens of *Providencia* spp. (Yuan et al., 2020) and occurs naturally in soil, water and sewage (Clifford et al., 2012). As a common clinical isolate, *P. stuartii* can cause urinary tract infections (UTIs) and other nosocomial infections in humans, such as pneumonia, meningitis, endocarditis, and wound and bloodstream infections (Keane et al., 1975; O'Hara et al., 2000; Armbruster et al., 2014; Kurmasheva et al., 2018). Additionally, the role of *P. stuartii* as a nosocomial pathogen in the dissemination of plasmid-mediated resistance has been confirmed (Franceschini et al., 1998; O'Hara et al., 2000). Currently, 15 *Providencia* species are recognized: *Candidatus P. siddallii, P. alcalifaciens, P. burhodogranariea, P. entomophila, P. heimbachae, P. huaxiensis, P. rettgeri, P. rustigianii, P. sneebia, P. stuartii, P. thailandensis, P. vermicola, P. friedericiana, P. manganoxydans* and *P. wenzhouensis*.^2^

In this work, we report a novel chromosome-encoded ANT gene, designated *aadA36*, in the strain *P. stuartii* P14, which was isolated from a sputum specimen from a burn patient. Whole-genome sequencing, genetic context analysis and kinetic parameter analyses were performed to characterize the molecular features of the *aadA36* gene and its related sequences.

## Materials and methods

### Bacterial strains and plasmids

A total of 25 *Providencia* isolates were collected from a hospital in Wenzhou, China, of which *P. stuartii* P14 was obtained from the sputum specimen of a burn patient. Species identification of these isolates was conducted by the VITEK 2 Compact instrument (bioMerieux, Inc., Craponne, France), 16S rRNA gene homology comparison and average nucleotide identity (ANI) analyses. The strains and plasmids used in this work are listed in Table 1.

**Table 1:** Bacteria and plasmids used in this work.

Strain or plasmid	Relevant characteristic(s)	Reference or source
**Strain**		
P14	The wild-type strain of *Providencia stuartii* P14	This study
DH5α	*Escherichia coli* DH5α was used as a host for cloning the *aadA36* gene	Our laboratory collection
BL21	*Escherichia coli* BL21 was used as a host for expression of the *aadA36* gene	Our laboratory collection
ATCC 25922	*Escherichia coli* ATCC 25922 was used as quality control for antimicrobial susceptibility testing	Our laboratory collection
pUCP20-*aadA36*/DH5α	DH5α carrying the recombinant plasmid pUCP20 -*aadA36*	This study
pCold I-*aadA36*/BL21	BL21 carrying the recombinant plasmid pCold I-*aadA36*	This study
**Plasmid**		
pUCP20	Cloning vector for the PCR products of the *aadA36* gene with its upstream promoter region, AMP^r^	Our laboratory collection
pCold I	Expression vector for the PCR products of the ORF of the *aadA36* gene, AMP^r^	Our laboratory collection

r, Resistance; AMP, Ampicillin.

### Antimicrobial susceptibility testing

The minimum inhibitory concentration (MIC) was determined using the agar dilution method following the guidelines of the Clinical and Laboratory Standards Institute (CLSI), and the susceptibility pattern was interpreted as described by the CLSI M100 (31st Edition, 2021) and the European Committee on Antimicrobial Susceptibility Testing (version 11.0, 2021). *Escherichia coli* ATCC 25922 was used as a reference strain for quality control. pUCP20/DH5α was used as the control strain for investigating the activity of the *aadA36* gene. The MIC experiment was performed on Mueller-Hinton (MH) agar plates with 2-fold serial dilutions of the antimicrobials, incubating the plates at 37°C for 20 h. The resistance breakpoint for florfenicol was determined according to a previous publication for *E. coli* (Wasyl et al., 2013), and the values for spectinomycin and streptomycin were interpreted according to the criteria proposed by the US FDA and the publications by Jouybari MA (Hu et al., 2017; Jouybari et al., 2021), respectively. All the tests were performed in triplicates.

### Whole-genome sequencing and functional analysis

Bacterial genomic DNA was extracted using the Generay Genomic DNA Miniprep Kit (Shanghai Generay Biotech Co., Ltd., Shanghai, China). Whole-genome sequencing was achieved using the Illumina NovaSeq (for all isolates) and PacBio RS II (only for an isolate to obtain a complete genome) platforms by Shanghai Personal Biotechnology Co., Ltd. (Shanghai, China). The short reads from Illumina sequencing of each isolate were assembled by MEGAHIT v1.2.9 (Li et al., 2016). To obtain a complete genome sequence for a certain isolate, the PacBio long reads were initially assembled using Trycycler v0.5.1 (Wick et al., 2021) and Flye v2.9-b1768 (Lin et al., 2016), and the quality of the draft genome assembly was further corrected with the short reads from Illumina sequencing by Pilon v1.24 (Walker et al., 2014). The open reading frames (ORFs) were then predicted using Prokka v1.14.6 (Seemann, 2014) and annotated by DIAMOND v2.0.11 (Buchfink et al., 2021) against the NCBI non-redundant protein database. The promoter region was characterized based on genome sequence information using BPROM.^3^ The resistance genes were annotated by Resistance Gene Identifier v5.2.0 (RGI)^4^ based on CARD (McArthur et al., 2013). ANI was computed using FastANI v1.33 (Jain et al., 2018). Multiple sequence alignment and neighbor-joining phylogenetic tree construction were performed using MAFFT v7.487 (Katoh and Standley, 2013) and IQ-TREE v 2.0.7 (Tamura et al., 2021), respectively. Plasmid prediction was performed by PlasmidFinder 2.0.^5^ Linear representation and visualization of the gene maps were achieved through genoPlotR v0.8.9 (Guy et al., 2010) and GView Server (Petkau et al., 2010), respectively.

### Molecular cloning of the resistance gene

The ORF of the predicted resistance gene with its promoter region (−155 bp) was amplified by PCR and then ligated into the pUCP20 vector with a T4 DNA ligase cloning kit (Takara Bio, Inc., Dalian, China). The recombinant plasmid was transformed into *E. coli* DH5α by the calcium chloride method, and then the transformants were cultured on Luria-Bertani (LB) agar plates supplemented with 100 μg/ml ampicillin. The inserted sequence in the recombinant was verified by Sanger sequencing. The primers used in this work are listed in Table 2.

**Table 2:** Cloning primers used in this study.

**Primer** ^**a**^	**Sequence (5′-3′)** ^**b**^	**Restriction endonuclease**	**Vector**	**Annealing temperature (°C)**	**Amplicon size(bp)**
pro-*aadA36*-F	ACAATGTAATAGACCGATCGGTCTGA		pUCP20	52	1,068
pro-*aadA36*-R	TTACTTTGCAAGCCGCTCTTCACAT		pUCP20		
orf-*aadA36*-F	GGATCCCTGGTGCCGCGCGGCAGCGTGTATGATCCTGTTGATAAAATTAA	*Bam*HI + Thrombin	pCold I	55	876
orf-*aadA36*-R	AAGCTTTATAAACGCCATAGAAATGATTTTTTTGAGTTTTTA	*Hind*III	pCold I		

a Primers with “pro” were used to clone the aadA36 gene with its promoter region, and primers with “orf” were used to clone the ORF of the aadA36 gene.

b The underlined sequences represent the restriction endonuclease sites.

### Expression and purification of recombinant AadA36

To obtain the AadA36, the ORF of the *aadA36* gene was amplified by PCR and then inserted into the pCold I vector between the cleavage sites of the restriction enzymes *Bam*HI and *Hind*III (Qing et al., 2004). The resultant recombinant plasmid pCold I-*aadA36* was introduced into *E. coli* BL21 competent cells. The transformants (pCold I-*aadA36*/BL21) were selected on LB agar plates supplemented with 100 μg/ml ampicillin. The presence of the *aadA36* gene in the recombinant strain was confirmed by PCR and Sanger sequencing of the PCR product (Shanghai Sunny Biotechnology Co., Ltd., Shanghai, China).

The overnight culture of the recombinant strain (pCold I-*aadA36*/BL21) was added to LB broth su pplemented with ampicillin (at a final concentration of 100 μg/ml) at a ratio of 1:100 for further incubation at 37°C in a shaker at 250 rpm. To induce the expression of AadA36, sterile isopropyl-beta-D-thiogalactopyranoside (IPTG) was added to the broth at a final concentration of 1 mM when the OD_600_ of the culture reached 0.6, as detected by ultraviolet–visible spectrophotometry, and then continue cultivating for 24 h at 15°C. Cells were harvested by centrifugation (8,000 × g, 10 min) at 4°C, resuspended in 3 ml of non-denaturing lysis buffer and disrupted by sonication for 5 min. The recombinant protein was purified using the BeyoGold His-tag Purification Resin and subsequently eluted with the nondenaturing eluent (50 mM NaH_2_PO_4_, 300 mM NaCl, 50 mM imidazole) from the His-tag Protein Purification Kit (Beyotime, Shanghai, China) according to the manufacturer’s instructions. The His-tag was removed from the samples using thrombin for 24 h at 37°C. Confirmation of the presence of AadA36 was obtained by sodium dodecyl sulfate-polyacrylamide gel electrophoresis (SDS-PAGE) and subsequent staining with Coomassie Brilliant Blue. The protein concentration was determined spectrophotometrically using a BCA protein assay kit (Beyotime, Shanghai, China).

### Enzyme kinetic studies of AadA36

The kinetic parameters of AadA36 were determined as reported previously with slight modifications (Kim et al., 2006). AadA36 activity was measured by coupling the enzymatic reaction to the UDP-glucose pyrophosphorylase, phosphoglucomutase and glucose-6-phosphate dehydrogenase reactions. The ANT(3″) catalytic activity was assayed by monitoring the accumulation of NADPH at 340 nm with a Synergy™ Neo2 Multi-Mode Microplate Reader (BioTek Instruments, Inc., United States). The reaction mixtures contained 50 mM HEPES (pH 7.5), 10 mM MgCl_2_, 0.2 mM UDP-glucose, 0.2 mM glucose 1,6-bisphosphate, 0.2 mM NADP, 0.2 mM dithiothreitol (DTT), 2 units/ml UDP-glucose pyrophosphorylase, 20 units/ml phosphoglucomutase, 20 units/ml glucose-6-phosphate dehydrogenase, 1 mM ATP, 8.8 × 10^−8^ μM purified AadA36, and variable concentrations of an aminoglycoside (5–150 μM) in a total volume of 0.2 ml. Reactions were initiated by the addition of the purified AadA36 enzyme. The steady-state kinetic parameters (*k_cat_* and *K*m) were determined by nonlinear regression of the initial reaction rates with the Michaelis–Menten equation in Prism (v9.4.0) software (GraphPad Software, CA, United States; Chen et al., 2019).

### Nucleotide sequence accession numbers

The nucleotide sequences of the chromosome, two plasmids (pP14-166 and pP14-114) of *P. stuartii* P14 and the *aadA36* gene have been deposited in GenBank under accession numbers NZ_CP097380, NZ_CP097381.1, NZ_CP097382.1 and ON520657, respectively.

## Results

### Identification of candidate novel resistance genes

Based on the annotation of the antimicrobial resistance genes (ARGs) in the genome sequences of the 25 isolates sequenced in this work, we screened the potential novel resistance genes that shared identities of <80% with the functionally characterized resistance genes, of which 9 hypothetical aminoglycoside resistance-related genes were chosen for further functional analysis. These genes included the *aac(6′)-Iy-*, *ant(9)-Ia-*, *aadA10-*, *aac(6′)-If-*, *aac(6′)-Iz-*, *ant(3″)-IIb-*, *aac(6′)-Iaa-*, *aac(6′)-Il-* and *aac(6′)-Isa-*like genes. Through preliminary *in vitro* antimicrobial susceptibility testing, among the recombinants with these cloned genes, we found that the *aadA10*-like gene (with the highest identity of 48.61% with the functionally characterized resistance genes, finally designated *aadA36* in this work) was functional. Of the 25 isolates sequenced in this work, the gene was found to be encoded in the P14 genome alone, and its molecular characteristics were subsequently analyzed.

### Characteristics and general features of *Providencia stuartii* P14 genome

The isolate P14 shared the closest relationship (97.14% coverage and 99.73% identity) with *P. stuartii* ATCC 29914 (NR_024848) based on 16S rRNA gene sequence analysis. Further analysis of the ANI revealed that it shared the highest identity (99.24%) with *P. stuartii* ATCC 33672 (NZ_CP008920.1). This was consistent with the clinical laboratory identification of the VITEK 2 Compact instrument. Therefore, this isolate was finally named *P. stuartii* P14.

The whole genome of *P. stuartii* P14 consisted of one chromosome that was 4,375,942 bp in length, encoding 3,967 coding sequences (CDSs) with an average GC content of 41.3% (Table 3). It contained two plasmids that were 166,313 bp and 114,876 bp in length, designated pP14-166 and pP14-114, respectively. A total of 19 resistance genes with ≥80% similarities with the functionally characterized ARGs were identified in the whole genome. These genes conferred resistance to 8 classes of antimicrobials: aminoglycosides [*aac(2′)-Ia*, *aph(6)-Id*, *aph(3″)-Ib*, *aac(6′)-Ib*, *ant(3″)-IIa*, *aph(3′)-Ia*], β-lactams (*bla*_CMY-2_ and *bla*_OXA-10_), fluoroquinolones (*rsmA* and *qnrVC4*), phenicols (*catIII*, *floR* and *cmlA5*), tetracyclines [*tet(B)* and *tet(A)*], sulfonamides (*sul2*), quinolones (*qacL*) and macrolides (*dfrA14* and *mphA*). Among them, *rsmA*, *tet(B)*, *catIII* and *aac(2′)-Ia* were located on the chromosome, and the other 15 resistance genes were on the plasmid pP14-166. Furthermore, the *in vitro* antimicrobial susceptibility test showed that the isolate P14 was resistant to 16 of the 25 tested antimicrobials, including aminoglycosides (spectinomycin, streptomycin, neomycin, ribostamycin, tobramycin, gentamicin, kanamycin and paromomycin), β-lactams (ampicillin, cefazolin, cefotaxime and ceftazidime), tetracycline, chloramphenicols (chloramphenicol and florfenicol), and nalidixic acid. In addition, the strain was susceptible to some other antimicrobials, such as amikacin, meropenem, and aztreonam. The MICs of the 25 antimicrobials and their corresponding resistance genes are shown in Table 4.

**Table 3:** General features of the *Providencia stuartii* P14 genome.

	**Chromosome**	**pP14-166**	**pP14-114**
Size	4,375,942	166,313	114,876
GC content (%)	41.3	52.5	40.4
Predicted coding sequences (CDSs)	3,967	200	130
Known proteins	2,636	54	48
Hypothetical proteins	1,331	146	82
Protein coding (%)	97.47	100	100
Average ORF length(bp)	907	737	705
Average protein length(aa)	306	245	234
tRNAs	80	0	0
rRNA operons	(16S-23S-5S) × 6, (16S-23S-5S-5S) × 1	0	0

**Table 4:** MICs of 25 antimicrobials for 5 strains(μg/ml).

Antimicrobial class	Chromosome-encoded related resistance gene	Plasmid-encoded related resistance gene	Antibiotics	ATCC 25922	DH5α	pUCP20/DH5α	pUCP20-*aadA36/*DH5α	*Providencia stuartii* P14
Aminoglycosides	*aac(2′)-Ia*	*ant(3″)-IIa,aph(6)-Id, aph(3″)-Ib, aph(3′)-Ia, aac(6′)-Ib*	Spectinomycin	8	8	8	1,024	512
			Streptomycin	4	2	2	128	64
			Neomycin	1	1	1	1	32
			Sisomicin	0.25	0.25	0.25	0.25	2
			Ribostamycin	2	2	2	2	512
			Tobramycin	0.25	0.25	0.25	0.25	32
			Gentamicin	0.25	0.25	0.5	0.25	16
			Amikacin	1	1	1	1	0.5
			Kanamycin	1	1	1	1	64
			Paromomycin	2	2	2	1	128
			Micronomicin	0.25	0.25	0.25	0.25	8
β-Lactams		*bla*_CMY-2_ *, bla*_OXA-10_	Ampicillin	4	2	/	/	512
			Cefoxitin	4	4	/	/	16
			Cefazolin	1	1	/		128
			Cefotaxime	0.125	0.125	/	/	16
			Ceftazidime	0.25	0.125	/	/	16
			Meropenem	0.03	0.03	/	/	0.06
			Aztreonam	0.125	0.03	/	/	2
Quinolones	*rsmA*	*qacL，qnrVC4*	Nalidixic acid	4	4	/	/	32
			Levofloxacin	0.03	0.03	/	/	1
Tetracyclines	*tet(B)*	*tet(A)*	Tetracycline	2	2	/	/	128
			Tigecycline	0.25	0.5	/	/	8
Phosphonic acid derivatives			Fosfomycin	2	2	/	/	2
Chloramphenicols	*catIII*		Chloramphenicol	4	4	/	/	128
		*floR, cmlA5*	Florfenicol	4	8	/	/	512
Trimethoprim and sulfonamides^a^		*sul2*	/	/	/	/	/	/
Macrolides^a^		*mphA, dfrA14*	/	/	/	/	/	/

a The antimicrobials with corresponding genes carried by the chromosome and plasmid pP14-166 but the susceptibility test was not performed.

### *aadA36* confers resistance to spectinomycin and streptomycin

The *aadA36* gene is 843 bp in length and encodes a 280 amino acid protein with a molecular mass of 31.74 kDa and a pI value of 5.03. The antimicrobial susceptibility results of the recombinant strain (pUCP20-*aadA36*/DH5α) showed that it conferred resistance to spectinomycin and streptomycin, with the MIC levels of both antimicrobials increasing 128-fold and 64-fold, respectively, compared with that toward the control strain (pUCP20/DH5α; Table 4). The kinetic parameters of AadA36 were consistent with the MIC data. The enzyme specifically adenylates spectinomycin and streptomycin with *k_cat_*/*K_m_* ratios of (1.07 ± 2.23) × 10^4^ M^−1^ s^−1^ and (8.96 ± 1.01) × 10^3^ M^−1^ s^−1^, respectively. As expected, no adenosine transfer was detected with tobramycin. The steady-state kinetic parameters for AadA36-catalyzed reactions are summarized in Table 5.

**Table 5:** Kinetic parameters of various aminoglycoside antimicrobials for purified AadA36.

**Substrate**	**kcat(s** ^**−1**^ **)**	Km(μM)^**a**^	**kcat/Km(M** ^**−**^ **1 s** ^**−1**^ **)**
SPE	0.506 ± 0.028	49.13 ± 12.84	(1.07 ± 2.23) × 104
STR	0.524 ± 0.066	59.52 ± 14.08	(8.96 ± 1.01) × 103
TOB	NA	NA	NA

SPE, spectinomycin; STR, streptomycin; TOB, tobramycin.

a Values are means ± standard deviations.

NA, no adenylate transfer activity was detected.

### Homology analysis of the novel aminoglycoside nucleotidyltransferase AadA36

**Figure 1:**
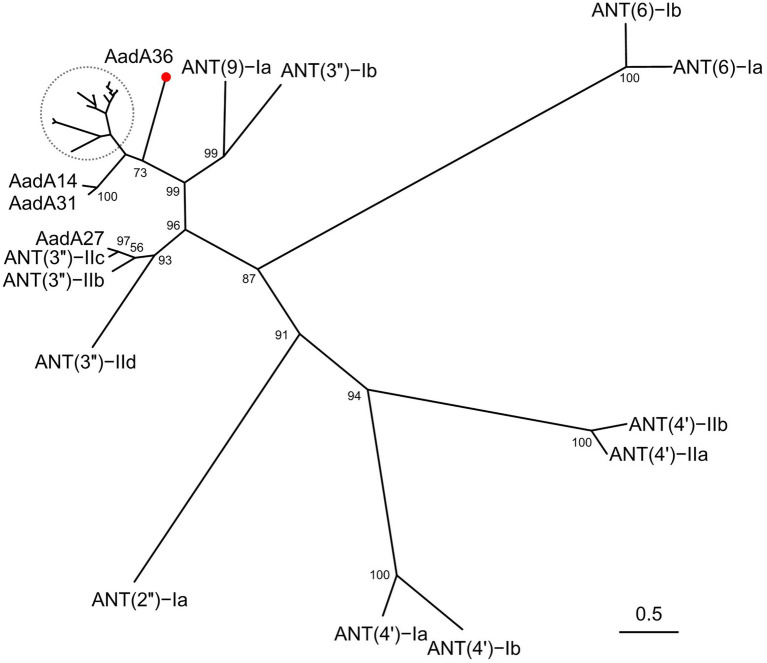
A phylogenetic tree showing the relationship of AadA36 with other functionally characterized aminoglycoside nucleotidyltransferases. AadA36 from our study is highlighted with a red dot. The dashed area includes AadA1, AadA4, AadA5, AadA6, AadA7, AadA8, AadA9, AadA10, AadA11, AadA13, AadA17, AadA21, AadA23, AadA24, AadA25, AadA28, AadA29, AadA30 and ANT (3″)-IIa. Bootstrap values were displayed at the nodes.

**Figure 2:**
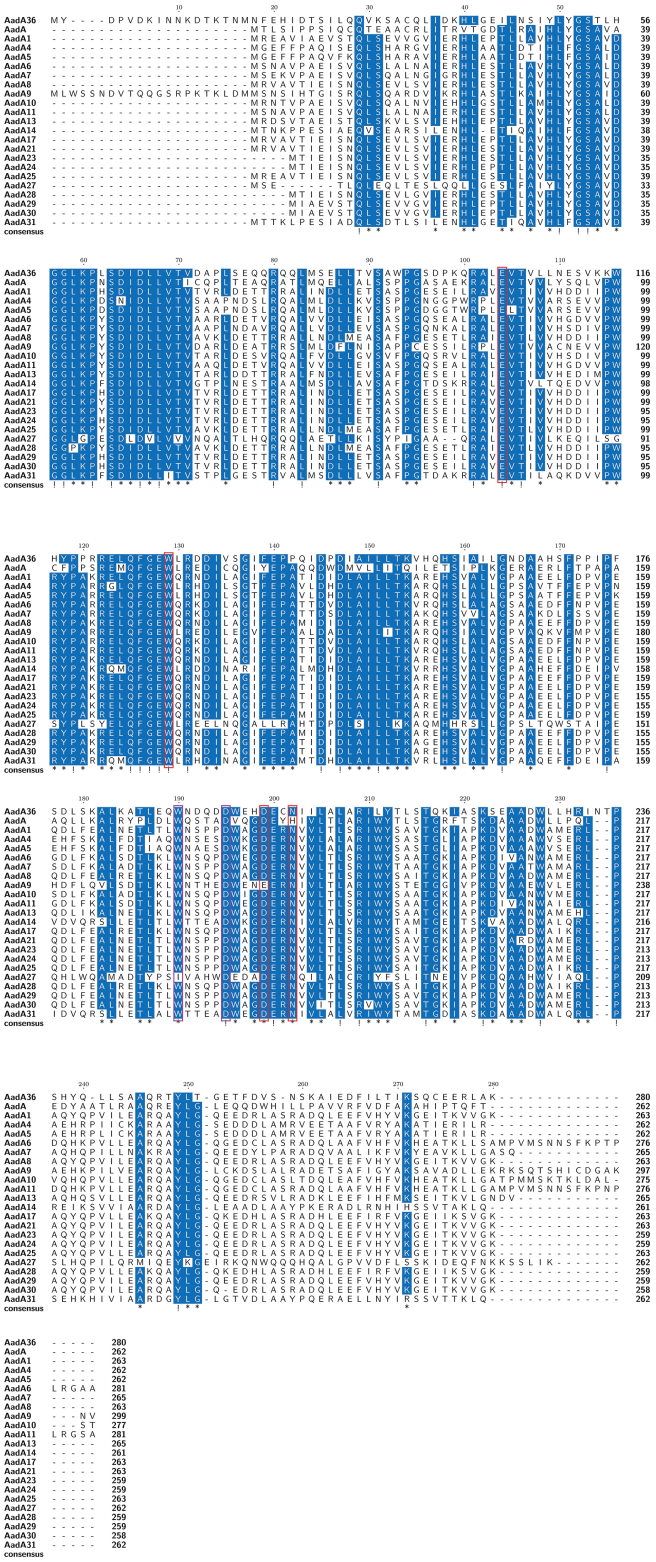
Multiple sequence alignment of the amino acid sequences of AadA36 and its relatives. The sequences and their accession numbers are as follows: AadA1 (AAO49597.1), AadA4 (AAN34365.1), AadA5 (AAF17880.1), AadA6 (CAJ32504.1), AadA7 (BAD00739.1), AadA8 (AAN41439.1), AadA9 (ABG49324.1), AadA10 (AAL36430.1), AadA11 (AAV32840.1), AadA13 (ABW91178.1), AadA14 (CAI57696.1), AadA17 (ACK43806.1), AadA21 (AAN87151.1), AadA23 (CAH10847.1), AadA24 (ABG72894.1), AadA25 (AET15272.1), AadA27 (CTQ57092.1), AadA28 (ANN23979.1), AadA29 (ANN23976.1), AadA30 (ANN23985.1), AadA31 (AUX81654.1) and AadA (Q8ZPX9.1). Exclamation marks indicate fully conserved residues, and asterisks indicate strongly similar residues. The red and purple frames indicate functional residues for spectinomycin and streptomycin, respectively. The numbers on the right represent the corresponding sequence length.

To determine the phylogenetic relationship of AadA36 with the ANTs, all 34 functionally characterized ANTs were collected from the CARD and the NCBI database. These included members of the five classes of the ANT family, including AadA14, AadA31, AadA10, AadA11, AadA13, ANT(6)-Ib, ANT(6)-Ia, ANT(2″)-Ia, ANT(4′)-Ia, ANT(4′)-Ib, and ANT(9)-Ia. The corresponding phylogenetic tree confirmed that the closest relative of AadA36 was AadA14 from *Pasteurella multocida* (51.92% identity and 83.57% coverage), followed by two AadA31 proteins, one from *Pasteurella multocida* and the other from *Histophilus somni* (both with an identity of 51.28% and coverage of 83.57%). This result suggested that AadA36 could be a novel lineage of the ANT (3″)-Ia family (Figure 1). To gain insight into the resistance function-related structural mechanism of AadA36, multiple sequence alignments of the deduced amino acid sequences of AadA36 and the other 22 sequences of ANT(3″)-Ia enzymes were performed. Among them, AadA6 (CAJ32504.1, 47.29% identity and 92.14% coverage), AadA10 (AAL36430.1, 48.61% identity and 89.64% coverage), AadA11 (AAV32840.1, 47.29% identity and 92.14% coverage), AadA14 (CAI57696.1, 51.92% identity and 83.57% coverage) and AadA31 (AUX81654.1, 51.28% identity and 83.57% coverage) shared relatively higher similarities (>42.8%) with AadA36. The results revealed that AadA36 contains six amino acid residues (E104, W129, D199, N202, W190 and D195; Figure 2).

### Analysis of the genetic context of the *aadA36* gene

**Figure 3:**
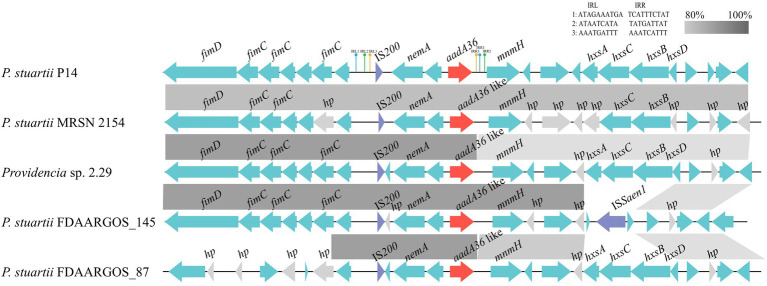
Comparative analysis of the genetic environment of the *aadA36* gene-related region. The *aadA36* homologous genes in different species were compared. Genes are denoted by arrows. The *aadA36* and *aadA36*-like genes were colored red, and the IS200 and ISSaen1 were colored purple. Gray shading denotes regions of homology (>80% nucleotide sequence identity), and hypothetical proteins (hp) are colored gray. The accession numbers of the sequences are as follows: *Providencia sp*. 2.29 (CP065420.1), *P. stuartii* MRSN 2154 (CP003488.1), *P. stuartii* FDAARGOS_145 (CP014024.2) and *P. stuartii* FDAARGOS_87 (CP031508.1).

The *aadA36* gene was located in the chromosome of *P. stuartii* P14. To gain insight into the genetic environment of *aadA36*, the region (approximately 21 kb in length) including the ORF of *aadA36* along with the approximately 10 kb upstream and downstream sequences was used as a query to search the NCBI non-redundant nucleotide database. A comparative genomic analysis of the *aadA36* encoding region with those of homologous sequences in three other *P. stuartii* strains and one *Providencia* sp. strain showed that the IS*200-nemA*-*aadA36-mnmH*-encoding fragment in the *P. stuartii* P14 chromosome exhibited high similarity with its relatives, and the fragment was flanked by two pairs of 9-bp imperfect inverted repeats (IRs) and one pair of 10-bp imperfect IRs (Figure 3). IS*200* in this work was an imperfect insert sequence encoding an 85-amino-acid transposase that shared 97.6% amino acid sequence identity with a transposase (AIN64626.1) of the IS*200*-like family. The finding indicates that this novel resistance gene might be transferable. To determine the origin of the novel resistance gene, more genomes of bacteria from different sources should be sequenced.

## Discussion

In this work, we identified a novel chromosome-encoded ANT gene designated *aadA36* from a clinical *P. stuartii* isolate P14. Of the 11 aminoglycoside antimicrobial agents tested, the recombinant strain (pUCP20-*aadA36*/DH5α) showed resistance only to streptomycin and spectinomycin. Phylogenetic analysis revealed that AadA36 is distantly related to the other AadA proteins. The closest relatives among the functionally characterized resistance genes were the genes encoding AadA14 and AadA31 of the ANT (3″)-Ia family, which shared amino acid sequence identities of less than 55%. The MIC values of *aadA36* to spectinomycin (1,024 μg/ml) and streptomycin (128 μg/ml) was roughly consistent with the genes of the *ant(3″)-Ia* family, such as *aadA14* (≥512 and 256 μg/ml), *aadA31* (>512 and 256 μg/ml), *aadA7* (>64 and > 64 μg/ml) and *aadA25* (≥512 and ≥ 64 μg/ml; Ahmed et al., 2004; Kehrenberg et al., 2005; Michael et al., 2012; Cameron et al., 2018).

Different ANTs have different aminoglycoside substrates. ANT(3″) adenylates streptomycin and spectinomycin (but not tobramycin; Kehrenberg et al., 2005), while ANT(6) confers resistance to streptomycin (Abril et al., 2010), and ANT(9) mediates resistance to spectinomycin (Kanchugal and Selmer, 2020). ANT(4′) and ANT(2″), however, confer resistance to multiple antimicrobials, including tobramycin (Wiedemann and Husing, 1985; Semper et al., 2020). Therefore, AadA36 could be distinguished from the other four subclasses of the ANT family and was assigned as a novel lineage of the ANT(3″)-Ia family.

The structural mechanism of AadA (Q8ZPX9) has been verified that the determinants for adenylation activity on streptomycin were amino acid residues W173 and D178, and on spectinomycin were E87, W112, D182, and 185H/N. Besides, the last four residues were conserved in all ANT (3″)(9) and ANT(9) enzymes (Stern et al., 2018). Multiple sequence alignments of AadA36 with the other AadA enzymes revealed that the six amino acid residues were conserved in them, except AadA9 (D203E) and AadA27 (W165I). Moreover, it has been reported that the structure of AadA31 was consistent with AadA_LT2_ and the residues implicated in ligand binding and catalysis was conserved within the active site (Cameron et al., 2018). It indicates that the mechanism of action of *aadA36* on streptomycin and spectinomycin may be related to these six amino acid residues.

The novel aminoglycoside resistance gene *aadA36* was related to a transposon-like sequence. The presence of the transposase gene upstream of the *aadA36* gene and three pairs of imperfect IRs flanking the *aadA36* encoding fragment demonstrated that the novel resistance gene carrying transposon-like sequence might be transferable. Furthermore, based on the amino acid sequence similarity analysis between AadA36 and other proteins retrieved from the database, we found that 21 proteins with identities >98% were all from the genus of *Providencia* (including 18 from *P. stuartii*), and all the other proteins showed identities of less than 74%. The results suggested that at present, the *aadA36* gene is conserved in species of the genus *Providencia*. The source and transmission mechanism of this novel resistance gene remain to be further studied.

The transposon-like structure related *aadA36* of this work was located in the chromosome, and the *ant(3″)-Ia* genes discovered so far have been found to be encoded on either plasmids or chromosomes, with many of them carried by the mobile genetic elements such as the class 1 integrons (Sandvang, 1999; Partridge et al., 2002; Michael et al., 2005). Overuse of antimicrobials exerts strong selective pressure on bacteria and facilitates the evolution and spread of drug-resistant strains. To some extent, strains carrying genes like *aadA36* can cope with the selection pressure of spectinomycin and streptomycin. On the other hand, the evolution of antimicrobial resistance may carry a fitness cost, in terms of reduced competitive ability when bacteria encounter an antimicrobial-free environment (Paulander et al., 2009). It has been found that chromosomal resistance mutations carry a larger cost than acquiring resistance *via* a plasmid (Vogwill and MacLean, 2015). However, due to its uncertain origin and the instability of compensatory evolution, the survival of strains carrying *aadA36* in the absence of antimicrobial selection pressure cannot be determined, which requires more efforts to verify.

## Conclusion

In this study, based on whole-genome sequencing, we characterized a novel chromosome-encoded ANT gene, *aadA36,* in a clinical *P. stuartii* isolate P14, which showed resistance to spectinomycin and streptomycin. Besides the chromosomal resistance genes, *P. stuartii* P14 also harbored a plasmid (pP14-166) encoding multidrug-resistance genes that conferred resistance to various antimicrobials, including aminoglycosides, β-lactams, tetracycline, and chloramphenicols. Sequence analysis revealed that the *aadA36* gene is related to a transposon-like sequence, which might indicate the possibility of transmission of this novel resistance gene between bacteria of different species. Identification of a novel resistance gene and characterization of its molecular characteristics will help us further elucidate the resistance mechanisms of clinical opportunistic pathogens and better cope with the corresponding infections.

## Data availability statement

The datasets presented in this study can be found in online repositories. The names of the repository/repositories and accession number(s) can be found in the article/Supplementary material.

## Ethics statement

Individual patient data was not involved, and only anonymous clinical residual samples during routine hospital laboratory procedures were used in this study. It was approved by the ethics committee of the Second Affiliated Hospital and Yuying Children’s Hospital of Wenzhou Medical University, Wenzhou, Zhejiang, China.

## Author contributions

JL, QB, and HZ conceived and designed the experiments. MG, YJ, WS, LZ, SL, AL, XZ, and QL performed the experiments. MG, CF, JL and QB data analysis and interpretation. MG, CF, QB, and HZ drafting of the manuscript. All authors contributed to the article and approved the submitted version.

## Funding

This study was supported by the Science & Technology Project of Wenzhou City, China (N20210001), Zhejiang Provincial Natural Science Foundation of China (LY19C060002 and LQ17H190001), and Natural Science Foundation of China (81973382).

## Conflict of interest

The authors declare that the research was conducted in the absence of any commercial or financial relationships that could be construed as a potential conflict of interest.

## Publisher’s note

All claims expressed in this article are solely those of the authors and do not necessarily represent those of their affiliated organizations, or those of the publisher, the editors and the reviewers. Any product that may be evaluated in this article, or claim that may be made by its manufacturer, is not guaranteed or endorsed by the publisher.

## Supplementary material

The Supplementary material for this article can be found online at: https://www.frontiersin.org/articles/10.3389/fmicb.2022.1035651/full#supplementary-material

SUPPLEMENTARY FIGURE S1 Multiple sequence alignment of the amino acid sequences of the AadA36 with other putative ANTs. The accession numbers are as follows: AMG65892.1, AXO17358.1, KNZ86850.1, KSX94677.1, MTC12158.1, QET96424.1, WP_004919927.1, WP_014658385.1, WP_040133143.1, WP_071821194.1, WP_071881681.1, WP_076913984.1, WP_102780545.1, WP_121875301.1, WP_141173405.1, WP_154610284.1, WP_154611447.1, WP_154625583.1, WP_174822199.1, WP_213537665.1, WP_223854774. Exclamations indicate fully conserved residues; asterisks indicate strongly similar residues. The numbers on the right represent the corresponding sequence length.

SUPPLEMENTARY FIGURE S2 A phylogenetic tree showing the relationship of AadA36 with other putative ANTs. AadA36 is highlighted with a red dot. The three columns on the right represent accession numbers, the taxonomy of the bacteria, and amino acid identity (%) with AadA36.

SUPPLEMENTARY FIGURE S3 SDS-PAGE of AadA36. Lane 1: PageRuler Prestained Protein Ladder (Thermo Fisher Scientific, product code: 26616); lane 2: uncleaved AadA36 with His6 tag; lane 3: cleaved AadA36 with thrombin.

## Footnotes

^1^ https://card.mcmaster.ca/ontology/36357

^2^ https://lpsn.dsmz.de/search?word=Providencia

^3^ http://www.softberry.com/berry.phtml?topic=bprom&group=programs&subgroup=gfindb

^4^ https://github.com/arpcard/rgi

^5^ https://cge.food.dtu.dk/services/PlasmidFinder-2.0/
