# Supplementary figures and images for: AadA36, a novel chromosomal aminoglycoside nucleotidyltransferase from a clinical isolate of Providencia stuartii
Source: Front Microbiol. 2022 Nov 1;13:1035651. doi: 10.3389/fmicb.2022.1035651 (PMC9663854; doi:10.3389/fmicb.2022.1035651)

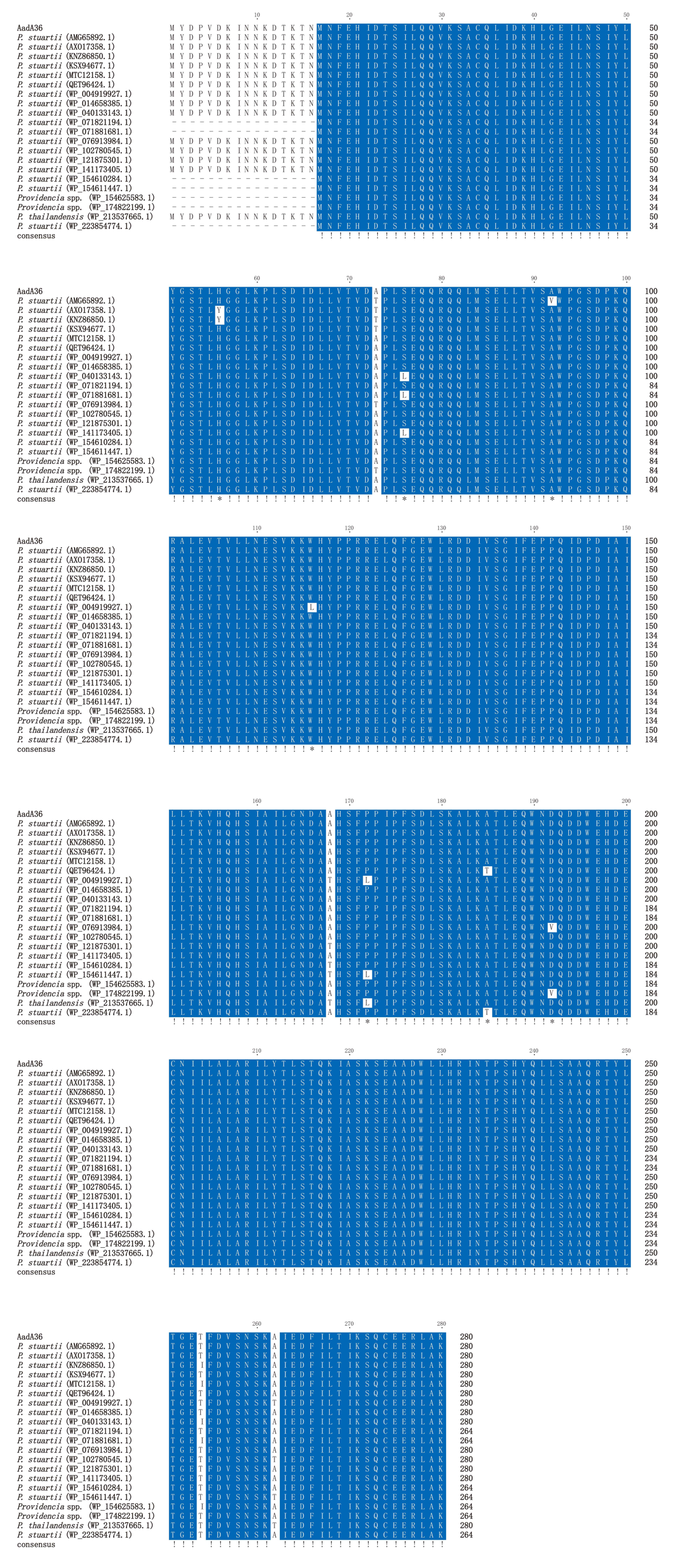

Supplement: SUPPLEMENTARY FIGURE S1 — Multiple sequence alignment of the amino acid sequences of the AadA36 with other putative ANTs. The accession numbers are as follows: AMG65892.1, AXO17358.1, KNZ86850.1, KSX94677.1, MTC12158.1, QET96424.1, WP_004919927.1, WP_014658385.1, WP_040133143.1, WP_071821194.1, WP_071881681.1, WP_076913984.1, WP_102780545.1, WP_121875301.1, WP_141173405.1, WP_154610284.1, WP_154611447.1, WP_154625583.1, WP_174822199.1, WP_213537665.1, WP_223854774. Exclamations indicate fully conserved residues; asterisks indicate strongly similar residues. The numbers on the right represent the corresponding sequence length. [file Image_1.JPEG]

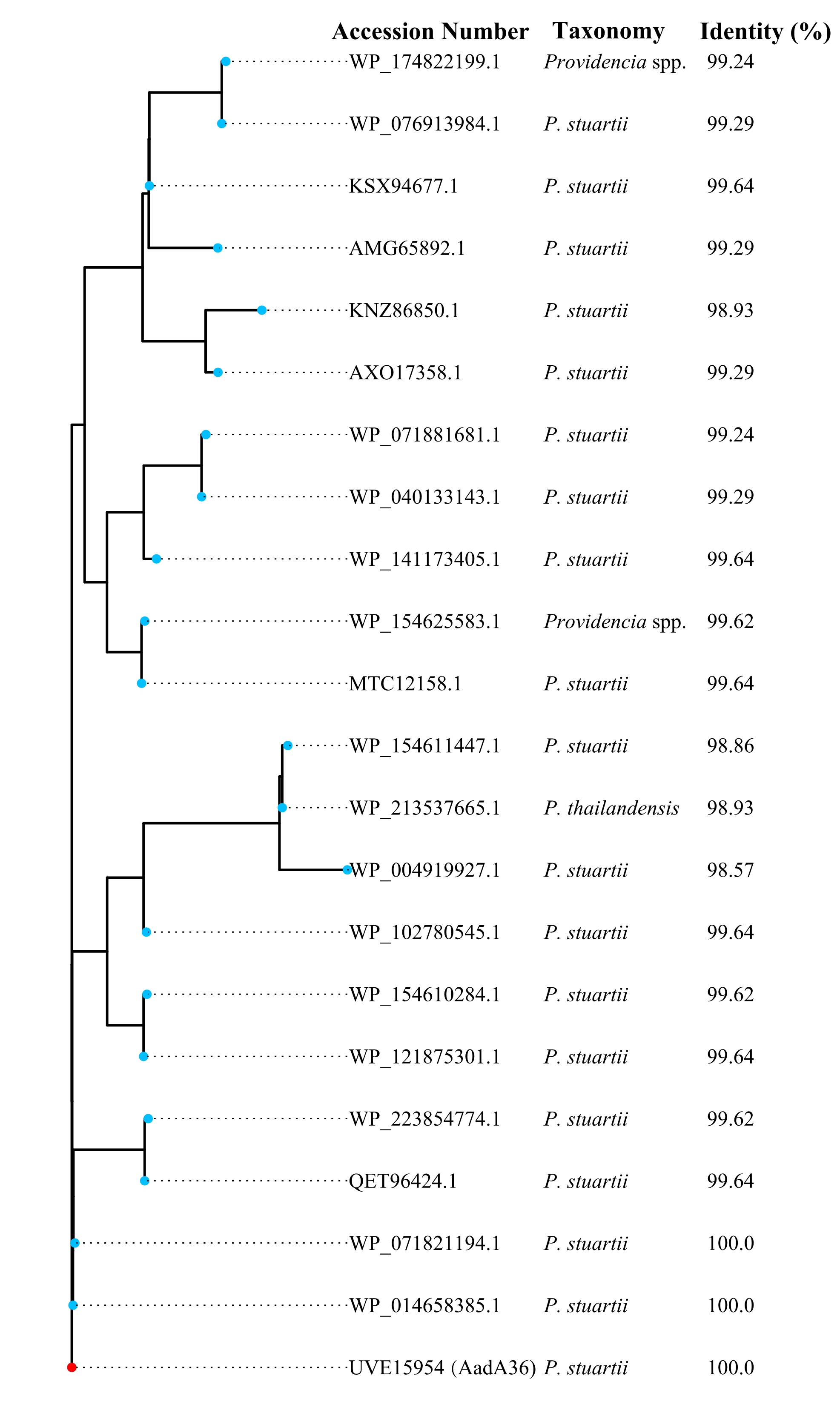

Supplement: SUPPLEMENTARY FIGURE S2 — A phylogenetic tree showing the relationship of AadA36 with other putative ANTs. AadA36 is highlighted with a red dot. The three columns on the right represent accession numbers, the taxonomy of the bacteria, and amino acid identity (%) with AadA36. [file Image_2.JPEG]

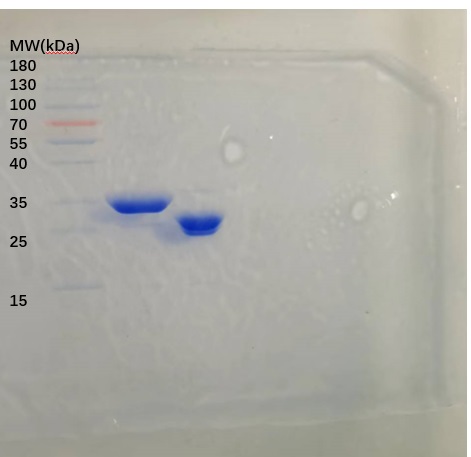

Supplement: SUPPLEMENTARY FIGURE S3 — SDS-PAGE of AadA36. Lane 1: PageRuler Prestained Protein Ladder (Thermo Fisher Scientific, product code: 26616); lane 2: uncleaved AadA36 with His6 tag; lane 3: cleaved AadA36 with thrombin. [file Image_3.JPEG]
